# Active ageing and self‐care in COVID‐19

**DOI:** 10.1002/nop2.1246

**Published:** 2022-06-05

**Authors:** Zahra Hatami, Hassan Barkati, Naeimeh Sarkhani, Nasrin Nikpeyma

**Affiliations:** ^1^ Department of Community Health and Geriatric Nursing, School of Nursing and Midwifery Tehran University of Medical Sciences Tehran Iran; ^2^ Nursing and Midwifery Care Research Center, School of Nursing and Midwifery Tehran University of Medical Sciences Tehran Iran

A novel virus from the coronavirus family called CoV‐2 SARS spread rapidly around the world and was considered a global health emergency. The COVID‐19 epidemic has severely affected the health of all human beings, especially older people (Pinto & de Carvalho, 2020). Studies show that the human coronavirus is an aggravating factor in the symptoms of diseases, such as obstructive pulmonary disease, asthma, heart failure, and underlying diseases in older people, which increases the need for acute emergency care and hospitalization. Therefore, paying attention to prevention and care behaviors such as self‐care helps to prevent infection and promote the health of these people (Jannat Alipoor & Fotokian, 2020). Also, due to the lack of definitive treatment for COVID‐19 and the unknown behavior of the virus, self‐care behaviors are the best possible solution to control this disease (Mehraeen et al., 2021).

Self‐care in older people is an active and practical process by which older people and their families help maintain health through health promotion and disease management measures (Matarese et al., 2018). Self‐care behaviours in COVID‐19 include (a) personal protective, (b) social distance, (c) disinfection of the environment, (d) psychological well‐being and (e) healthy lifestyle (De Maria et al., 2020). Self‐care behaviours affect COVID‐19 disease and if done correctly, the rate of hospitalization can be reduced by up to 60% (Zareipour et al., 2020). Self‐care behaviours help reduce the burden of health care providers, reduce treatment costs and improve the health of older people (Pourhabib et al., 2018). Older people’s self‐care behaviours in COVID‐19 are related to various factors such as previous experience of self‐care in other diseases, family support and health care organizations, type of job, physical and personality characteristics of the elderly person, flexibility, positive emotions, vitality, hope and optimism (Kooij, 2020). Because new mutations of the disease appear in different mutations of the COVID‐19 virus, so it is very important to pay attention to self‐care behaviours. However, staying at home can cause the elderly to experience symptoms such as anxiety and depression, limit their social relationships and significantly affect their lifestyle (Pant & Subedi, 2020). From the perspective of health scientists, one of the ways to improve the health of older people in the aspects of health and their participation in maintaining their health is to pay attention to active ageing, which can play an important role in preventing their disease (León et al., 2020), it is also associated with COVID‐19 self‐care behaviours.

Active ageing was introduced in 2002 by the World Health Organization with an emphasis on the culture and social conditions of society. According to the WHO, active ageing is a process that provides maximum opportunities for the health, participation, safety, and lifelong learning of older people to improve their quality of life (Del Barrio et al., 2018). In this definition, health emphasizes maintaining a healthy lifestyle and reducing risk factors and includes physical, mental and social well‐being. Participation refers to the optimization of opportunities related to the social environment, such as participation in social and charitable affairs and education. Security refers to activities designed to ensure the protection, dignity and attention to the physical, social and financial needs of older people (Bárrios, 2015; Del Barrio et al., 2018). From the perspective of older people, active ageing is a set of personal components, such as maintaining health, life satisfaction, establishing family and friendly relationships and adapting to changes related to ageing and self‐care (Bárrios, 2015). Some factors determine active ageing. These factors include health promotion and disease prevention, self‐care, culture and social conditions, behavioural (physical activity), psychological (cognitive impairment and depression), environmental (physical barriers and access to transportation) and economic factors (income, employment) and access to medicines and health services (León et al., 2020; Mohammadi et al., 2018). Ferreira et al. reported that there is a direct relationship between functional independence in activities, such as self‐care (eating, bathing, dressing), defecation control, mobility, communication and social interaction with active ageing (Ferreira et al., 2012). Souza et al. (2021) found that educational programs and health‐promoting activities contribute to the advancement of active ageing in the fields of social participation, interaction with health care providers, cognitive improvement, participation in physical activity and increasingly older people’s independence.

Ageing and physiological changes in the body, weak immune system, one or more chronic diseases and polypharmacy in older people make this group more at risk for COVID‐19 and its complications. One of the main methods of prevention and control of COVID‐19 disease in older people is self‐care. Active ageing is one of the indicators of health promotion and disease prevention in older people, so health care organizations must pay attention to active ageing as one of the factors related to self‐care behaviours, and design appropriate health programs to maintain the physical, mental, functional and social potentials of the older people so that, through active ageing, self‐care behaviours for diseases such as COVID‐19 can be performed, and the quality of life can be enhanced.

## AUTHOR CONTRIBUTIONS

Study conception: N.N, Z.H, H.B. Data collection: N.N, Z.H, N.S.H.B. Drafting and approving of the article: N.N, N.S.

## CONFLICT OF INTEREST

The authors declare that they have no competing interests.

## REFERENCES
